# Assessment of malaria treatment interventions: a critical analysis of government initiatives and causes of treatment failure at Port Loko Government Hospital, Sierra Leone

**DOI:** 10.1186/s12936-025-05330-9

**Published:** 2025-03-14

**Authors:** Lawrence Sao Babawo, Rashid Bundu Kpaka, Daniel Karim Dauda Sesay

**Affiliations:** 1 Faculty of Health Sciences and Disaster Management, Eastern Technical University of Sierra Leone, Kenema, Sierra Leone; 2 District Health Management Team (DHMT), Karene District, Port Loko, Sierra Leone

**Keywords:** Malaria, Treatment failure, Socio-economic determinants, Public health, Sierra Leone, Government interventions

## Abstract

**Background:**

Malaria remains a significant public health challenge, particularly in sub-Saharan Africa, where it accounts for high morbidity and mortality rates. Sierra Leone, including Port Loko District, continues to experience a high burden of malaria despite government interventions. This study evaluates the existing government interventions for malaria treatment at Port Loko Government Hospital, examining the socio-economic determinants contributing to malaria treatment failure.

**Methods:**

The study employed a cross-sectional study design with a quantitative approach, involving 150 health workers and 150 women of childbearing age. A purposive stratified random sampling technique was used to ensure diverse representation. Primary data were collected using structured questionnaires, while secondary data were extracted from the District Health Information System (DHIS) and hospital records. Data were analysed using SPSS version 28.0, applying descriptive statistics (frequencies, percentages, means and SD) and inferential analyses (Chi-square tests and binary logistic regression) to assess associations between socio-economic factors and treatment-seeking behaviours.

**Results:**

Findings revealed that 90% of health workers were aware of malaria interventions, but only 68% reported high adherence to treatment guidelines. Among women of childbearing age, 40% sought malaria treatment at drug stores or pharmacies due to financial constraints, and a significant association was observed between socio-economic status and healthcare-seeking behaviour (^2^ = 9.32, df = 4, p = 0.05). Despite the fact that 73.3% reported the use of long-lasting insecticidal nets (LLINs), malaria prevalence remained high, suggesting additional risk factors beyond bed net usage.

**Conclusion:**

This study highlighted persistent challenges in malaria control, including inadequate healthcare access, non-adherence to treatment regimens, and socio-economic constraints. Policy recommendations include strengthening malaria treatment adherence programmes, improving healthcare accessibility, and enhancing community-based malaria prevention strategies.

## Background

Malaria remains a significant public health concern in Sierra Leone and across sub-Saharan Africa, contributing to high morbidity and mortality rates, particularly among vulnerable populations. The disease disproportionately affects vulnerable members of the community including women of reproductive age, young children, and low-income communities, where access to healthcare services and preventive measures is often inadequate [1]. Despite global efforts to combat malaria, its persistence is exacerbated by socio-economic factors, limited healthcare infrastructure, and behavioural challenges to treatment adherence [2].

Globally, malaria leads to approximately 350–500 million clinical episodes annually, with mortality estimates ranging between 1 and 3 million deaths, predominantly affecting children in endemic countries [3, 4]. The World Malaria Report 2020 recorded 229 million malaria cases in 2019, with 409,000 deaths, highlighting the disproportionate burden on impoverished communities [5]. Despite efforts by the World Health Organization (WHO) to achieve malaria elimination by 2030, the disease persists, necessitating targeted interventions to improve prevention, diagnosis, and treatment access.

Regionally, sub-Saharan Africa (SSA) bears the highest malaria burden, accounting for nearly 95% of global cases and deaths. The region experiences an estimated 1.5–2.7 million malaria-related deaths annually. Contributing factors include favourable climatic conditions for mosquito breeding, the prevalence of the virulent *Plasmodium falciparum* species, and widespread poverty coupled with inadequate healthcare infrastructure [6]. Malaria is a leading cause of childhood mortality in SSA, with about 25% of deaths among children under five years attributed to the disease. Survivors of cerebral malaria often face long-term neurological complications, including blindness, epilepsy, and speech impairments, which further hinder their development [7].

In Sierra Leone, malaria remains endemic and accounts for a substantial burden of disease. The 2016 Sierra Leone Malaria Indicator Survey (SLMIS) estimated that malaria contributes to 40.3% of outpatient morbidity across all age groups and 47% among children under five. Furthermore, malaria is responsible for 37.6% of hospital admissions, with a case fatality rate of 17.6% (Ministry of Health and Sanitation Sierra Leone) [8]. To mitigate the impact of malaria, the Government of Sierra Leone, through the National Malaria Control Programme (NMCP), has implemented various interventions, including the adoption of artemisinin-based combination therapy (ACT), the distribution of long-lasting insecticidal nets (LLINs), indoor residual spraying, and public health campaigns emphasizing environmental sanitation and mosquito exposure reduction [9]. While these interventions have improved access to preventive measures, numerous challenges remain, including frequent stock-outs of essential malaria drugs, inadequate healthcare infrastructure, poor adherence to treatment, and persistent socio-economic determinants, such as poverty and low education levels [10]. Moreover, poor environmental sanitation and inadequate utilization of LLINs exacerbate the malaria burden, highlighting the urgent need for targeted interventions [2]. Socio-economic determinants play a crucial role in malaria treatment failure where poverty restricts individuals' ability to access healthcare services, purchase essential medications, and implement preventive measures, such as the use of LLINs and indoor residual spraying [11]. Education significantly influences treatment-seeking behaviours, with lower literacy levels often correlating with non-adherence to medical recommendations [12].

Port Loko District is located in the North-West part of Sierra Leone and has one of the highest malaria prevalence rates in the country. The high incidence is primarily driven by socio-economic factors such as poverty, inadequate and impoverished healthcare infrastructure, including shortages of trained personnel, medical supplies, and diagnostic tools, low literacy levels, and unfavourable health-seeking behaviours [13]. These constraints contribute to treatment delays, misdiagnosis, and suboptimal patient outcomes, reinforcing the need for a holistic and multi-sectoral approach to malaria control [14]. Addressing these socio-economic factors is essential to enhancing the effectiveness of malaria control programmes, reducing disease burden, and improving health outcomes for affected communities.

This study, therefore, evaluates the effectiveness of existing government malaria interventions in Port Loko District, with a specific focus on identifying key causes of malaria treatment failures, assessing socio-economic determinants influencing treatment-seeking behaviours, and proposing evidence-based policy recommendations for improved malaria management.

## Theoretical framework

The theoretical framework for this study is based on the Health Belief Model (HBM) and the Social Determinants of Health (SDH) Theory, which are commonly applied in public health research related to disease prevention and management, particularly in the context of malaria control.

The Health Belief Model (HBM) posits that individuals are more likely to take preventive actions or seek treatment for a health condition when they perceive that they are at risk, believe the health behaviour will reduce that risk, and feel that the benefits of the action outweigh the perceived barriers [15]. In the context of malaria in Port Loko District, the model can help explain why certain individuals or communities may not seek timely treatment or preventive measures like bed nets, despite the widespread availability of these interventions. HBM can be used to assess the perceived severity of malaria, susceptibility to the disease, and the perceived benefits and barriers to taking preventive actions such as adherence to treatment and environmental management.

Social Determinants of Health (SDH) theory recognizes that factors such as socio-economic status, access to healthcare, education, housing, and environmental conditions have a profound impact on health outcomes. According to the World Health Organization [16], social determinants influence a population’s vulnerability to diseases like malaria, which can be exacerbated by poor access to healthcare, inadequate housing conditions, and low socioeconomic status. This theory is particularly relevant in Port Loko District, where poverty, poor infrastructure, and limited access to healthcare may contribute to both malaria prevalence and the barriers to effective treatment.

## Conceptual framework

The conceptual framework for this study revolves around a multi-dimensional approach to understanding malaria management and its determinants in Port Loko District. The framework incorporates both individual and contextual factors influencing malaria prevention, treatment-seeking behaviour, and treatment failure.

The study assesses the effectiveness of government interventions such as the distribution of bed nets, indoor spraying with insecticides, and availability of anti-malarial drugs. These interventions are expected to reduce malaria prevalence if they are consistently available, accessible, affordable and effective [17]. However, failure in the implementation or inconsistent supply may contribute to treatment failures.

Socio-economic factors, such as income level, education, and employment status, significantly influence treatment-seeking behaviours. Studies have shown that individuals from higher socio-economic backgrounds are more likely to seek professional medical care and adhere to prescribed malaria treatments regimens [18]. In contrast, low-income individuals may rely on informal sources like drug shop and/or traditional sources that will potentially lead to ineffective treatment [19].

The accessibility and quality of healthcare services play a crucial role in malaria treatment success. Barriers such as long distances to healthcare facilities, inadequate trained and qualified healthcare workers, and stock-outs of malaria treatment commodities often contribute to treatment failures [20]. This framework suggests that effective malaria control requires not only adequate supply chains for treatment commodities but also improved healthcare infrastructure, such as mobile clinics and community health workers.

Environmental risk factors such as poor sanitation and inadequate housing are critical in the transmission of malaria. This study will explore how environmental factors like stagnant water, poor waste management, and lack of mosquito control measures (e.g., drainage systems and clean water supply) increase the malaria burden in Port Loko District [21].

**Fig. 1:**
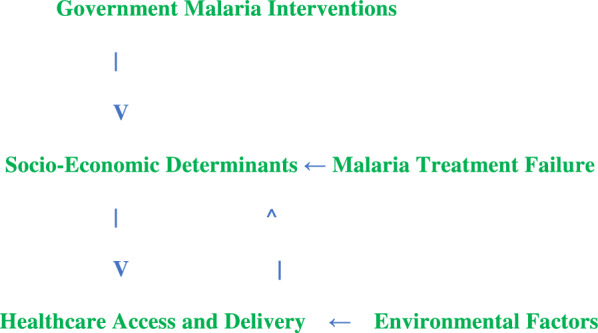
Conceptual framework

Malaria treatment failure is a key outcome of interest in this study. This can result from several factors, including incorrect diagnosis, substandard medications, incomplete treatment courses, or the emergence of drug-resistant malaria strains [17]. This conceptual framework links these factors with government interventions, socio-economic determinants, and access to healthcare services (Fig. 1).

## Methods

### Study design, sampling method

This study was conducted at Port Loko Government Hospital, Bekeh Loko Chiefdom, Port Loko District, using a cross-sectional study design with a quantitative research approach. The cross-sectional design was chosen as it allowed data collection at a single point in time, making it suitable for assessing the effectiveness of malaria interventions, identifying causes of treatment failure, and evaluating socio-economic factors influencing treatment-seeking behaviours. This approach was cost-effective, time-efficient, and provided a comprehensive snapshot of the malaria management landscape in Port Loko District.

To ensure a well-structured and representative sample, a purposive stratified random sampling technique was employed. This method was chosen to capture key stakeholders directly involved in malaria treatment and prevention. Two distinct population groups were identified such as healthcare workers and women of childbearing age. Health workers (n = 150) were included because of their critical role in malaria diagnosis, treatment, and prevention. Their perspectives on government interventions, adherence to treatment protocols, and challenges in malaria management provided valuable insights into healthcare service delivery. Meanwhile, women of childbearing age (n = 150) were selected due to their heightened vulnerability to malaria infection and their role as primary caregivers for children under five, another high-risk group. Their healthcare-seeking behaviours, knowledge of malaria prevention, and adherence to treatment were essential factors in understanding the socio-economic determinants contributing to malaria treatment failure.

Participant selection followed a two-stage process. First, a purposive selection targeted healthcare workers actively engaged in malaria management, including medical doctors, nurses, midwives, and laboratory technicians. It also focused on women aged 15–49 years who regularly accessed healthcare services at the hospital. Following this, a stratified random sampling approach was applied within each group to minimize selection bias and ensure diverse representation across different professional cadres and demographic backgrounds. This method helped to achieve a balanced sample, ensuring that the findings accurately reflected the real-world experiences and challenges of malaria management in Port Loko District.

### Research tool, data collection methods

Data for this study were obtained from both primary and secondary sources. Primary data were gathered through a structured survey questionnaire administered to health workers and women of childbearing age. The questionnaire was carefully designed to capture key areas relevant to malaria management. It included demographic information such as age, level of education, occupation, and household income, which provided a contextual understanding of the respondents. The questionnaire also assessed knowledge, attitudes, and practices (KAP) related to malaria, focusing on awareness of prevention methods, adherence to treatment protocols, and perceptions of healthcare services.

To evaluate the effectiveness of government malaria interventions, the survey examined access to ACT, rapid diagnostic tests (RDTs), and other initiatives led by the NMCP. It also identified the major barriers to effective malaria treatment, including drug stock-outs, poor adherence to treatment regimens, delays in seeking care, and socio-economic constraints that limit access to healthcare services. Furthermore, the study investigated the utilization of preventive measures such as the use of LLINs, environmental sanitation practices, and exposure to malaria-related health education campaigns.

The questionnaire consisted of a yes or no closed ended question, Likert-scale items, and a few open-ended questions, allowing respondents to provide detailed insights where necessary. To ensure the reliability and internal validity of the research instrument, it underwent pre-testing and was assessed for internal consistency using Cronbach’s alpha coefficient formula. The analysis yielded a Cronbach’s alpha value of 0.89, indicating high reliability and confirming that responses across different sections of the questionnaire were consistent. To further assess internal consistency, validity and reliability, triangulation of results with other studies was also done.

In addition to primary data, secondary data were extracted from the District Health Information System (DHIS) and health facility registers at Port Loko Government Hospital. These records provided an objective assessment of malaria trends and healthcare performance over time. The secondary data included malaria case records, tracking incidence and prevalence rates over the past five years, and hospital admission and outpatient attendance data, particularly disaggregated by age to highlight the burden of malaria among children under five and pregnant women.

Stock out reports and the availability of anti-malarial drugs were also reviewed to track shortages of essential malaria treatment supplies, including ACT and RDTs. Additionally, the study analysed adherence to treatment regimens and outcomes, especially the proportion of patients who completed full ACT regimens and documented cases of treatment failure. To assess preventive strategies, secondary data on the coverage and utilization of malaria prevention measures were examined, including the distribution and uptake of LLINs, indoor residual spraying campaigns, and seasonal malaria chemoprevention efforts.

### Data processing and statistical analysis

To ensure accuracy and robustness in addressing the study objectives, the collected data were subjected to a two-step structured statistical analysis process. The raw data were first entered and cleaned using Microsoft Excel 2019, where inconsistencies, missing values, and duplicate entries were identified and corrected. Categorical variables were appropriately coded, while continuous variables were standardized where necessary to maintain consistency. Once the quality checks were completed, the cleaned dataset was exported into Statistical Package for Social Sciences (SPSS) version 28.0 for a more comprehensive statistical analysis.

A combination of descriptive and inferential statistical techniques was employed to analyse the data, ensuring that the findings aligned with the study objectives. To summarize and interpret key trends in the dataset, frequencies and percentages were computed for categorical variables such as the distribution of respondents by gender, occupation, malaria knowledge, and awareness of malaria interventions. For continuous variables like age, income levels, and time taken to seek treatment, measures of central tendency including means and standard deviations were used to provide a clear representation of the data. Additionally, visualizations such as frequency tables, bar charts, and histograms were generated to enhance the clarity of findings and support better interpretation.

Inferential statistical techniques were applied to explore relationships between different variables, Chi-square (χ^2^) tests were used to assess associations between categorical variables, i.e. relationship between socio-economic factors and treatment-seeking behaviour. A binary logistic regression analysis was conducted to identify significant predictors examining factors influencing malaria treatment adherence and the effectiveness of interventions. All statistical tests were conducted at a 95% confidence interval, (p < 0.05) ensuring statistical significance.

## Results

A combination of descriptive and inferential statistical techniques was employed to analyze the data, ensuring alignment with the study objectives.

### Descriptive statistical analysis

#### Health workers’ characteristics (n = 150)

The mean age of health workers is 34.5 years, with an average of 8.2 years of experience, indicating a relatively young and active workforce. The majority of health workers (90.0%) are aware of government malaria interventions, but only 68.0% report high adherence to treatment guidelines (Table 1).

**Table 1:** Health Workers’ demographic characteristics

Variable	Mean ± SD/frequency (%)
Age (years)	34.5 ± 7.8
Gender	Male: 72 (48.0%)
	Female: 78 (52.0%)
Occupation	Nurses: 85 (56.7%)
	CHOs: 35 (23.3%)
	Lab Technicians: 30 (20.0%)
Years of Experience	8.2 ± 5.4 years
Malaria Knowledge Level	High: 95 (63.3%)
	Moderate: 40 (26.7%)
	Low: 15 (10.0%)
Awareness of Government Malaria Interventions	Yes: 135 (90.0%)
	No: 15 (10.0%)
Adherence to Treatment Guidelines	High: 102 (68.0%)
	Moderate: 35 (23.3%)
	Low: 13 (8.7%)

#### Women of childbearing age characteristics (n = 150)

The mean age of women of childbearing age is 28.6 years, with 60.0% married and a moderate level of formal education among the majority. 40.0% (n = 60) of women of childbearing age seek treatment at a drug store as their first point of contact when they or a household member falls ill which may indicate limited healthcare access or financial constraints. Additionally, 37.7% (n = 57) visit the nearest health facility, while 5.0% (n = 15) seek care elsewhere. Despite 73.3% reporting bed net use, malaria prevalence remains high, suggesting additional environmental or behavioral risk factors (Table 2).

**Table 2:** Women of Childbearing Age Characteristics (n = 150)

Variable	Mean ± SD / Frequency (%)
Age (years)	28.6 ± 6.2
Marital Status	Married: 90 (60.0%)
	Single: 45 (30.0%)
	Divorced/Widowed: 15 (10.0%)
Educational Level	No formal education: 20 (13.3%)
	Primary: 55 (36.7%)
	Secondary: 50 (33.3%)
	Tertiary: 25 (16.7%)
Average Monthly Income (SLL)	870,000 ± 350,000 SLL
Healthcare-Seeking Behavior	Health Facility: 57 (37.7%)
	Drug Store: 60 (40.0%)
	Traditional Healer: 18 (12.0%)
	Others: 15 (10.0%)
Malaria Prevention Knowledge	High: 80 (53.3%)
	Moderate: 50 (33.3%)
	Low: 20 (13.3%)
Household Use of Bed Nets	Yes: 110 (73.3%)
	No: 40 (26.7%)

From Table 3, analysis of healthcare-seeking behaviour is critical for evaluating malaria treatment accessibility. Inferential analysis using chi-square tests (χ^2^ = 9.32, df = 4, p = 0.05) revealed a significant association between socioeconomic status and healthcare-seeking behaviour, with lower-income individuals more likely to seek treatment from drug stores rather than formal healthcare facilities.

**Table 3:** Observed frequencies of healthcare-seeking behaviour by socioeconomic status

Socioeconomic status	Formal healthcare facility	Drug store	Traditional healer	Total
Low Income	30	25	10	50
Middle Income	23	18	10	51
High Income	30	13	8	51
Total	**68**	**56**	**28**	**150**

The bold value signifies the importance of identifying the different statuses as related to the socio economic variables of healthcare-seeking behaviour

### Factors contributing to high malaria prevalence

Identifying key factors driving malaria prevalence is essential for targeted interventions. Table 4 reveals that 41.0% (n = 62) of women attributed high malaria prevalence to multiple factors, including poor sanitation, substandard housing, lack of access to healthcare, and not sleeping under bed nets. Additionally, 33.7% (n = 51) identified poor environmental sanitation as a primary factor, while only 2.7% (n = 4) cited hunger as a contributing factor.

**Table 4:** Factors responsible for high malaria prevalence

Factors	Frequency	Percentage (%)
Poor environmental sanitation	51	33.7
Not Sleeping under a bed net	10	6.7
Lack of access and availability of health services	8	5.0
Sub-standard housing	17	11.0
Hunger	4	2.7
All of the above	62	41.0
Total	**150**	**100.0**

The bold value signifies the importance of identifying the different statuses as related to the socio economic variables of healthcare-seeking behaviour

### Most prevalent disease conditions among pregnant women attending ANC

As shown in Table 4, 73.3% (n = 110) of health workers reported that malaria is the most common condition among ANC attendees. Other conditions included iron deficiency anaemia (13.3%, n = 20), typhoid fever (8.3%, n = 12), and pre-eclampsia (4.3%, n = 6). Malaria remains a leading health concern among pregnant women attending antenatal clinics (ANC), emphasizing the critical need for routine malaria screening and preventive measures during pregnancy (Table 5).

**Table 5:** Most prevailing disease conditions of pregnant women that attend a clinic

Disease conditions	Frequency	Percentage (%)
Malaria	110	73.3
Iron deficiency	20	13.3
Pre-eclampsia	6	4.3
Typhoid	12	8.3
Others	1	0.7
Total	**150**	**100.0**

### Binary logistic regression results

The outcome variable (dependent variable) is malaria prevalence (1 = high prevalence, 0 = low prevalence). Independent variables include poor sanitation, lack of healthcare access, and other contributing factors. The binary logistic regression analysis above showed that Poor environmental sanitation (OR = 2.45, p < 0.05) significantly increased the risk of malaria prevalence. Women who reported poor sanitation were 2.45 times more likely to experience malaria compared to those in cleaner environments highlighting the need for community-based interventions. Lack of access to healthcare (OR = 2.89, p < 0.05) also had a significant effect. Women with limited healthcare access were 2.89 times more likely to report high malaria prevalence. Not sleeping under bed nets (OR = 1.85, p = 0.072) showed a positive association but was not statistically significant at the 5% level. Hunger (OR = 1.12, p = 0.408) was not significantly associated with malaria prevalence, (Table 6).

**Table 6:** Binary logistic regression analysis

Predictor variable	Odds ratio (OR)	95% CI	p-value
Poor Environmental Sanitation	2.45	(1.50–3.98)	0.003
Lack of Access to Healthcare	2.89	(1.73–4.82)	0.001
Not Sleeping Under Bed Nets	1.85	(0.95–3.60)	0.072 (Not Significant)
Hunger	1.12	(0.40–2.78)	0.408 (Not Significant)

### Factors contributing to malaria treatment failure

Understanding malaria treatment failure is critical for improving patient outcomes. Table 7 shows that 93.3% (n = 140) of health workers identified non-adherence to prescribed treatment as the leading cause of malaria treatment failure. Other contributing factors included presumptive treatment (63.0%, n = 95), drug overuse (53.0%, n = 80), and the use of sub-therapeutic drugs (72.0%, n = 108).

**Table 7:** Frequency and percentage distribution of responses showing the factors responsible for malaria treatment failure

Parameters	Frequency			
Factors responsible for the ineffective malaria treatments	Yes	No	% Yes	% No
Non-adherence to prescribed treatment	140	10	93.3	6.7
Presumptive treatment	95	55	63.0	37.0
Drug overuse	80	70	53.0	47.0
Use of sub-therapeutic drugs	108	42	72.0	28.0

Using the observed frequencies from Table 7, the chi-square statistic was calculated as χ^2^ = 20.0, df = 2, p < 0.05) confirms a significant association between education level and adherence to prescribed malaria treatment. Patients with secondary or tertiary education exhibited the highest adherence rate (83.3%), whereas those with no formal education were the least adherent (33.3%). This suggests the need for targeted health education interventions to improve medication adherence and treatment success rates.

### Availability of malaria diagnosis, treatment, and prevention commodities

**Fig. 2:**
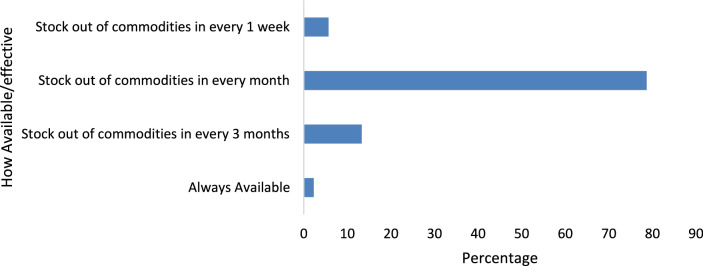
Availability and adequacy of commodities for complicated and uncomplicated malaria Diagnosis and treatment

The availability of essential malaria commodities is crucial for effective disease management. Figure 2 indicates that 85.0% (n = 128) of health workers experience monthly stock outs of malaria commodities, while only 5.0% (n = 8) reported consistent availability of diagnostic kits and treatment supplies (Fig. 2).

Using the observed frequencies in (Fig. 2), a chi-square test of independence was applied to determine if commodity availability is significantly associated with treatment delays. The chi-square test (χ^2^ = 12.87, df = 2 p = 0.002) confirms a statistically significant association between frequent stock outs and treatment delays, emphasizing the critical need for a robust supply chain to improve malaria case management. Frequent stockouts (85.0% of health workers) were strongly associated with treatment delays, with 105 out of 128 reporting delays. Occasional stockouts showed a moderate impact, with 7 out of 15 reporting delays. Consistent availability of malaria commodities was linked to fewer delays, with only 2 out of 8 experiencing treatment delays**.**

### Perception of malaria diagnosis and treatment effectiveness

As shown in Table 8, 78.0% (n = 117) of health workers perceived malaria commodities as only partially effective, while 14.0% (n = 21) reported them as ineffective. Only 8.0% (n = 12) considered them highly effective. Assessing the effectiveness of available malaria diagnosis and treatment commodities is essential for ensuring optimal patient outcomes.

**Table 8:** Perception of how effective is the commodities for malaria diagnosis and treatment

How effective and adequate	Frequency	Percentage (%)
Highly effective	12	8
Partially effective	117	78
Not effective	21	14
Total	150	100

### Patient adherence by education level

Using the observed frequencies from Table 9, the chi-square statistic was calculated as χ^2^ = 20.0, df = 2, p < 0.05) confirms a significant association between education level and adherence to prescribed malaria treatment. Patients with secondary or tertiary education exhibited the highest adherence rate (83.3%), whereas those with no formal education were the least adherent (33.3%). This suggests the need for targeted health education interventions to improve medication adherence and treatment success rates.

**Table 9:** Observed frequencies of patient adherence by education level

Education Level	Adherent (n, %)	Non-Adherent (n, %)	Total (n, %)
No Formal Education	20 (33.3%)	40 (66.7%)	60 (40.0%)
Primary Education	30 (50.0%)	30 (50.0%)	60 (40.0%)
Secondary/Tertiary	25 (83.3%)	5 (16.7%)	30 (20.0%)
Total	**75 (50.0%)**	**75 (50.0%)**	**0 (100%)**

## Discussion

The findings of this study reveal that the majority of health workers in Port Loko District (90.0%) are aware of government malaria interventions, yet only 68.0% adhere strictly to treatment guidelines. This suggests a discrepancy between awareness of malaria interventions and adherence to prescribed guidelines which may be due to a number of factors, including inadequate resources, training gaps, or challenges in supply chain management, which could hinder the effective implementation of government programs. Previous research supports these findings; with studies indicating that knowledge alone is not sufficient to ensure proper treatment practices [22]. Interventions aimed at improving adherence to malaria treatment protocols need to address these systemic challenges to improve outcomes. Malaria control programmes should, therefore, integrate strategies to enhance not only awareness but also adherence. Strengthening the healthcare system's logistical capacity, especially in the supply of diagnostic kits and treatment commodities, will help mitigate stock outs, which were reported by 85.0% of health workers in this study. Continuous monitoring of stock availability and ensuring regular supply of anti-malarial drugs could enhance treatment efficacy and improve adherence rates.

The study also highlights significant socio-economic determinants affecting healthcare-seeking behaviour. It was found that 40.0% of women of childbearing age sought treatment from drug stores, a reflection of potential financial constraints or limited access to formal healthcare services. This is consistent with existing literature, where lower socio-economic status often correlates with reliance on informal healthcare providers [23]. This trend is particularly concerning because treatment at drug stores may not guarantee the same quality of care, diagnostic accuracy, or effectiveness of malaria treatment. The finding emphasizes the need for targeted interventions addressing accessibility barriers, particularly for lower-income populations. Expanding subsidized malaria treatments and outreach programs that increase awareness of government health services could reduce the reliance on informal providers. Programmes that promote the integration of malaria services into primary healthcare settings are likely to reduce this disparity.

Factors contributing to high malaria prevalence were identified, with 41.0% of women attributing it to poor environmental sanitation, substandard housing, and lack of access to healthcare. This aligns with the findings of other studies which have shown that environmental factors such as poor sanitation and housing conditions are significant drivers of malaria transmission [24]. Additionally, the 73.3% of women reporting the use of bed nets indicates that bed net coverage is still not sufficient to prevent malaria, suggesting that further action is needed to address environmental factors. Malaria control programmes should adopt a multi-sectoral approach that addresses both medical and environmental determinants of malaria. Government interventions should enhance campaigns promoting proper sanitation and housing improvements, alongside increasing bed net distribution and ensuring their correct usage. The integrated approach, as advocated by the World Health Organization (WHO), can lead to more sustainable malaria elimination efforts [25].

Despite the availability of malaria diagnosis and treatment commodities, 78.0% of health workers perceived these commodities as only partially effective raising concerns about the effectiveness of available treatments and diagnostics, with potential implications for treatment success. Diagnostic tools and anti-malarial drugs are essential components of malaria control, their effectiveness depends on correct handling, storage, usage and adherence to treatment protocols. A review of malaria diagnostic and treatment protocols is needed to identify gaps in effectiveness. Strengthening training for healthcare workers on the proper use of these commodities could help improve treatment outcomes as well as the proper handling and management of the malaria commodities supply chain and continuous monitoring of their effectiveness is very important.

The identification of non-adherence to prescribed treatment (93.3%) as the primary cause of malaria treatment failure is concerning, as it points to a fundamental issue in patient education and behavior. Previous research has shown that patient non-adherence is a critical factor in treatment failure and the development of drug resistance [26]. Presumptive treatment, drug overuse, and the use of sub-therapeutic drugs further exacerbate this issue. Malaria treatment policies must therefore emphasize the importance of patient education where health workers engage in more proactive health education campaigns to encourage adherence to treatment regimens. Equally improving diagnostic accuracy could help prevent the misuse of anti-malarial drugs through the reduction of presumptive treatment practices.

This study provides a critical analysis of the malaria situation in Port Loko, however, there are several limitations. The reliance on self-reported data may introduce bias, particularly regarding adherence to treatment guidelines and healthcare-seeking behaviour. Moreover, the study failed to explain the specific reasons for the high non-adherence rates, such as financial constraints or lack of trust in healthcare services, which could offer more specific policy recommendations. Furthermore, this study was conducted in a single district, and findings may not be generalizable to other areas with different socio-economic and healthcare characteristics. Future research should include a larger sample size and explore qualitative aspects of malaria treatment and prevention from the perspectives of patients and healthcare providers.

## Conclusion

This study highlights significant gaps in malaria control efforts in Port Loko District. To reduce malaria prevalence and improve treatment outcomes, malaria control programs must consider socio-economic factors, enhance healthcare accessibility, and address environmental risk factors. Moreover, strengthening adherence to treatment and improving the effectiveness of malaria commodities are critical areas for intervention. government malaria control programmes can be more responsive to the needs of the population and make substantial strides toward reducing malaria burden if policies are adapted that reflect these findings.

## Data Availability

The data presented in this study are available on request from the corresponding author upon reasonable request.
